# RETRACTION: Methanol Extract of *Ocimum Gratissimum* Protects Murine Peritoneal Macrophages from Nicotine Toxicity by Decreasing Free Radical Generation, Lipid and Protein Damage and Enhances Antioxidant Protection

**DOI:** 10.1155/omcl/9876960

**Published:** 2026-05-19

**Authors:** Oxidative Medicine and Cellular Longevity

RETRACTION: S. K. Mahapatra, S. P. Chakraborty, S. Das, and S. Roy, “Methanol Extract of Ocimum Gratissimum Protects Murine Peritoneal Macrophages from Nicotine Toxicity By Decreasing Free Radical Generation, Lipid and Protein Damage and Enhances Antioxidant Protection,” *Oxidative Medicine and Cellular Longevity* 2, no. 4 (2009): 1–9, https://doi.org/10.4161/oxim.2.4.9000.

The above article, published online on 13 May 2009 in Wiley Online Library (wileyonlinelibrary.com), has been retracted by agreement between the Journal Editor, Jeannette Vasquez‐Vivar; and John Wiley & Sons Ltd. The retraction has been agreed following an investigation into concerns raised by a third party and PubPeer comments [1] regarding image inconsistencies. Figure 3A shows evidence of identical duplicate macrophages within the image. Figure 3B shows evidence of having been constructed from multiple images, with residual straight lines and corners resulting from their overlap. Figure 3C shows evidence of identical duplicate macrophages within the image. The authors failed to respond to requests for explanations of the above. The concerns have resulted in the editor losing confidence in the results presented, therefore the article must be retracted.

The authors were informed of the retraction, but did not respond.
